# A luminescent aluminium salen complex allows for monitoring dynamic vesicle trafficking from the Golgi apparatus to lysosomes in living cells†
†Electronic supplementary information (ESI) available. See DOI: 10.1039/c7sc04498d


**DOI:** 10.1039/c7sc04498d

**Published:** 2018-01-08

**Authors:** Juan Tang, Hao-Yan Yin, Jun-Long Zhang

**Affiliations:** a Beijing National Laboratory for Molecular Sciences , State Key Laboratory of Rare Earth Materials Chemistry and Applications , College of Chemistry and Molecular Engineering , Peking University , Beijing 100871 , P. R. China . Email: zhangjunlong@pku.edu.cn ; Fax: +86-10-62767034

## Abstract

The Golgi apparatus is well-known as the center of vesicle trafficking whose malfunction might cause the breakdown of overall cellular architecture and ultimately cell death. The development of fluorescent probes to not only precisely stain the Golgi apparatus but also monitor dynamic vesicle trafficking is of great significance. While fluorescent proteins and fluorescent lipid analogs have been reported, they are sometime limited by either overexpression and toxicity or lack of high selectivity, respectively. We herein report a novel approach based on metal-induced coordination between the phosphate anions of phospholipids and the metal center of a luminescent Alsalen complex **AlL**, which can *in situ* track membrane vesicle trafficking from the Golgi apparatus to the lysosomes in living cells. This work opens a new avenue for designing luminescent metal probes based on the Lewis acidity of metal ions and allows the use of metal ions with different charge states, polarities, and reactivities within a similar structural scaffold to expand coordination chemistry for biological studies.

## Abstract 2

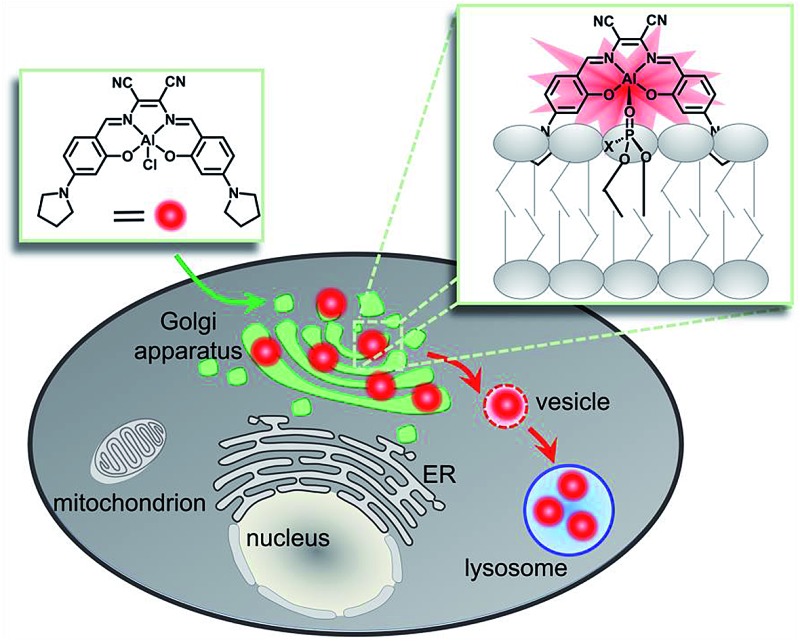
Tracking vesicle transport from the Golgi apparatus to lysosomes based on an Al^3+^–phospholipid coordination strategy.

## Introduction

The Golgi apparatus is well-known as a “post office” in live cells, and collects and modifies proteins and lipids from the endoplasmic reticulum (ER) and then transports them to other organelles including lysosomes *via* vesicle trafficking.^1^ Malfunction in vesicle transport may cause the breakdown of overall cellular architecture and ultimately cell death.^2^,^3^ Thus, it is important to visualize the dynamic intracellular processes of the Golgi apparatus.

**Scheme 1:**
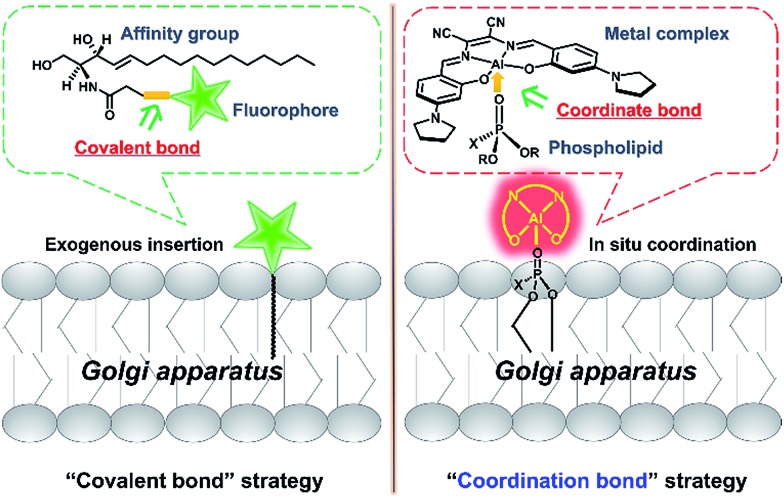
Schematic illustration of two strategies for tracking lipids. The “covalent bond” strategy: the insertion of an exogenous fluorophore–lipid covalent adduct. The “coordination bond” strategy: the *in situ* coordination of an endogenous lipid to a metal complex.

To achieve this goal, fluorescent probes are required to not only precisely stain the Golgi apparatus, but also monitor the vesicle trafficking pathways. To meet these requirements, fluorescent proteins have been used as intrinsic Golgi apparatus trackers, which play a critical role and have offered a lot of useful information in understanding the biology of the Golgi apparatus.^4^ However, this approach sometimes suffers from low cell transfection efficiency and false positive signals that can alter the cell phenotype and/or lead to oxidative damage.^4^ To address these issues, the development of small molecular probes specific to the Golgi apparatus is a promising approach,^5^ but few of them are able to monitor the dynamics of vesicle trafficking processes. For example, fluorescent lipid analogs by attaching lipids such as ceramide to organic fluorophores like BODIPY, have been used to track dynamic processes from the Golgi apparatus to the cell membrane surface based on the well-known metabolism similar to its endogenous counterparts (Scheme 1, left).^6^–^8^ Vesicle trafficking from the Golgi apparatus to lysosomes is another canonical transportation process of vital importance.^9^,^10^ However, small molecular fluorescent probes have rarely been reported to track such processes. We herein report an alternative approach, which depends on the coordination ability of metal complexes to precisely *in situ* bind to endogenous natural lipid components in the Golgi apparatus and can monitor intracellular vesicle trafficking to the lysosomes. Therefore, without the need for operation outside the cells such as the covalent attachment of lipid molecules to fluorophores, these luminescent metal complexes represent a convenient and easy approach to monitor the Golgi apparatus-centered vesicle trafficking.

Luminescent metal complexes are an emerging class of fluorescence bioprobe due to their prominent photophysical properties such as long lifetimes and large Stokes shifts *etc.*^11^–^28^ Another bonus of such probes, in comparison with organic fluorescent bioprobes, is the ability to use different metal ions to modulate the charge state, polarity, redox property and even reactivity within a similar structural scaffold to fine-tune the biological behaviors and functions. Despite their potential, few probes have been reported to take full advantage of these properties.^29^ Recently, several lipophilic luminescent metal complexes with positive charge have been reported to stain the Golgi apparatus.^30^–^32^ These pioneering studies have inspired us to replace the divalent Zn^2+^ ion in a neutral, ER specific Znsalen complex (salen = *N*,*N*′-bis(2-hydroxy-4-(pyrrolidin-1-yl)benzyl-idene)-1,2-dicyano-1,2-ethenediamine) with trivalent Al^3+^, which rates a Golgi apparatus-targeting cationic Alsalen complex, **AlL**.^33^ Cellular internalization studies showed that **AlL** was internalized into the Golgi apparatus *via* membrane vesicle trafficking along microtubules. Importantly, since the Al^3+^ ion has strong oxophilicity, **AlL** exhibit a high affinity to negatively mono-charged phospholipids with a binding constant up to 1.2 × 10^6^ M^–1^, which allows it to anchor to the membrane structure of the Golgi apparatus. More importantly, cell imaging showed that **AlL** could be preferentially transported to the Golgi apparatus, the distributing hub of vesicle transportation, then underwent vesicle-mediated transportation along microtubules, and further trafficked into the lysosomes. Therefore, this work provides a new access to designing luminescent metal probes based on the Lewis acid reactivity of metals (Scheme 1, right) to monitor the dynamics of biological events in living cells.

## Results and discussion

### Synthesis and characterization

Starting from 2-hydroxy-4-(pyrrolidin-1-yl)benzaldehyde, the Znsalen complex (salen = *N*,*N*′-bis(2-hydroxy-4-(pyrrolidin-1-yl) benzylidene)-1,2-dicyano-1,2-ethene diamine) was obtained by condensation with 1 equiv. 2,3-diaminomaleonitrile and 1 equiv. Zn(OAc)_2_. **AlL** was obtained *via* a transmetalation reaction of Znsalen and AlCl_3_ (1 equiv.) in a yield of 56%. **AlL** was characterized by high-resolution electrospray ionization mass spectroscopy (HR-ESI-MS), ^1^H NMR spectrometry, UV-vis absorption and IR spectroscopy (ESI†). As shown in Fig. S1,† **AlL** in DMSO showed a typical band centered at 394 nm with a low-energy shoulder at 443 nm and another band centered at 595 nm. Upon excitation at 390 nm, **AlL** displayed intense red emission centered at 643 nm with a quantum yield of 0.49 in DMSO (Table S1†). Similar to its Znsalen counterpart,^33^**AlL** exhibited a two-photon absorption cross section (*δ*) of *ca.* 180 GM at 840 nm, referring to rhodamine B (Fig. S2†), suggesting its potential application as a two-photon fluorescence probe.

**Scheme 2:**
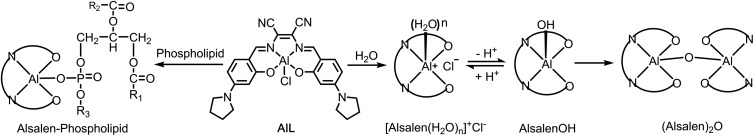
Speciation of **AlL** in water and its reactivity with phospholipids.

Due to the strong Lewis acidity of Al^3+^ and labile chloride binding, **AlL** tends to undergo hydrolysis in aqueous media (Scheme 2),^34^–^36^ transforming it into neutral [Alsalen(OH)] and therefore generating the μ-oxo dimer (Alsalen)_2_O.^37^ We monitored the hydrolysis process of **AlL** in HEPES buffer (pH 6.0, 10 mM) by UV-vis absorption spectroscopy. We assumed that the replacement of a chloride anion by a water ligand and formation of the (Alsalen)_2_O dimer happened faster than deprotonation of the bound water ligand, and thus the whole hydrolysis process could be simplified to obey first-order kinetics. Therefore the absorption maximum decay at 588 nm was fitted to first-order kinetics, which follows the equation below.

1 ln(*A*_0_/*A*) = *kt*

Plotting the logarithm of *A*_0_/*A versus* time (*t*) gave a linear relationship from 500–7000 s (Fig. S3†). The fitted hydrolysis rate constant *k* is 2.8 × 10^–5^ s^–1^.

We then studied the effect of the pH of **AlL** in Britton–Robinson buffer which has an adjustable pH ranging from 2.0 to 12.0. As shown in Fig. S4,† increasing the pH from 4.0 to 10.0 in Britton–Robinson buffer led to broadened UV-vis absorption of **AlL** and a progressively decreased absorption intensity. The nonlinear regression of the absorption centered at 588 nm *vs.* pH affords a p*K*_a_ value of 6.9 ± 0.1, which is confirmed by fluorescence titration. These results suggest that **AlL** acts mainly as a monomeric cationic complex in the Golgi apparatus (pH ∼ 6.5) or lysosomes (pH ∼ 5.0–6.0).^38^

### Selective binding to phospholipids

Aluminum cations (Al^3+^), known as hard Lewis acids, have strong oxophilicity and play an important role in many reactions such as the ring-opening polymerization of epoxides,^39^ Diels–Alder reaction,^40^ and Michael addition.^41^ Coordination of a phosphate ligand to an Al^3+^ complex, to form a neutral aluminophosphate with an Al–O–P linkage, has been extensively applied in molecular sieves, ion exchange resins, and adsorption media.^42^–^45^ However, this property has been seldom explored in biological applications, despite the fact that phosphate species widely exist in biological systems such as phospholipids and nucleic acids *etc*. To date, there are only two reports on using Alsalen to detect double stranded polynucleotides (polyG)^46^ and G-type nerve agents^47^ in aqueous solution.

**Fig. 1:**
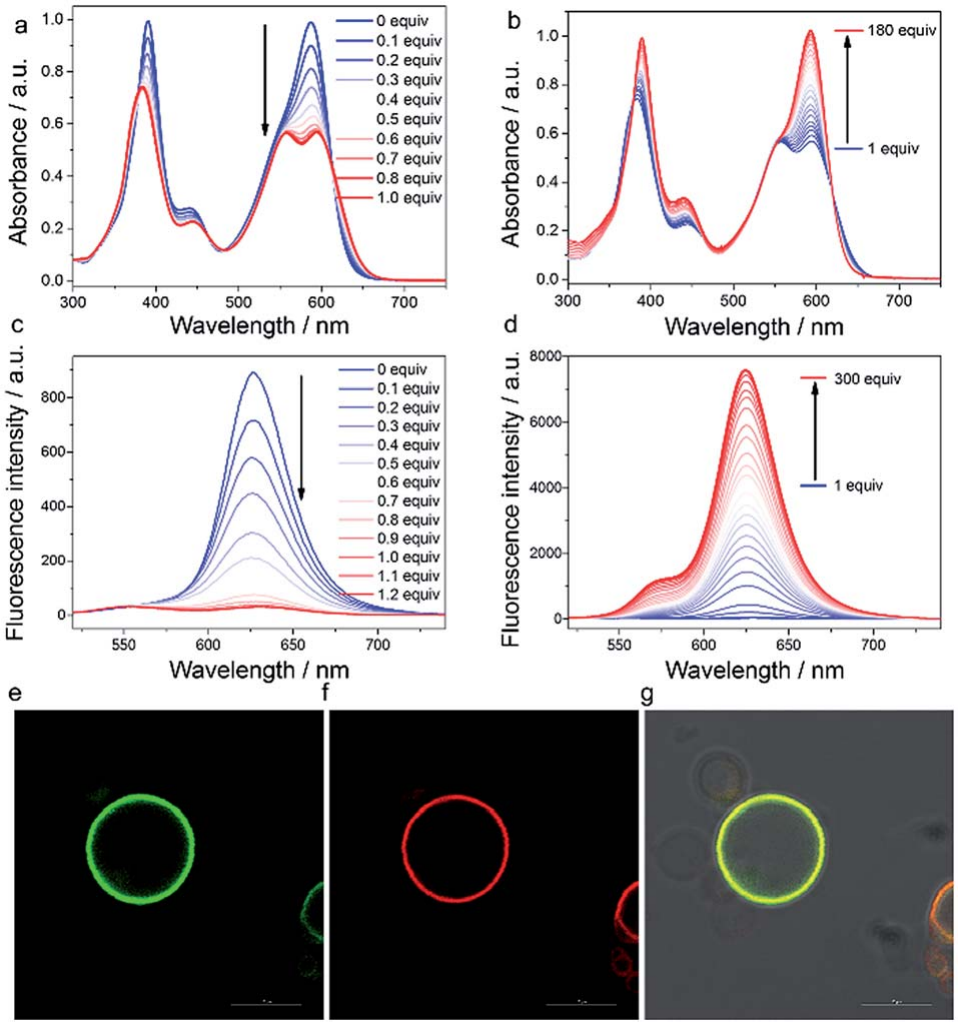
(a–d) Titration of **AlL** (20 μM) with PG in HEPES buffer (pH 6.0, 10 mM). (a and b) UV-vis and (c and d) fluorescence spectra, *λ*_ex_ = 415 nm. (e) Image of CF-PE; (f) image of **AlL**; (g) merged image of (e and f) and Differential Interference Contrast (DIC) images; scale bar = 10 μm.

To probe the binding abilities of **AlL**, we titrated **AlL** (20 μM) with several series of oxo-containing substrates including sulfates, sulfonates, carboxylates and phosphates in HEPES buffer (pH 6.0, 10 mM) at 25 °C and monitored the process by UV-vis absorption and fluorescence spectroscopy. The results are compiled in Table 1, Fig. 1 and Fig. S5–S8.† We found that only the oxo-containing lipids with one negative charge, such as sodium dodecyl sulphate (SDS), sodium dodecylbenzenesulfonate (SDBS), oleic acid (OA), phosphatidic acid (PA), phosphatidylglycerols (PG), 1,2-dioleoyl-*sn-glycero*-3-phosphoethanolamine-*N*-(carboxyfluorescein) (CF-PE) and 1-caproyl-2-[12-[(7-nitro-2-1,3-benzoxadiazol-4-yl) amino]dodecanoyl]-*sn-glycero*-3-phosphoethanolamine (NBD-PE), showed strong binding ability to **AlL**. Meanwhile, the neutral lipids (such as phosphatidylcholine (PC) and phosphatidylethanolamine (PE)), hydrophilic phosphates (such as mamose-6-phosphate (M6P) and ctDNA) and oxo-containing anions (such as H_2_PO_4_^–^, HCO_3_^–^ and SO_4_^2–^) did not. These titration experiments revealed that **AlL** can only bind to oxo-containing species that are mono-negatively charged and bear lipid structures. The above results also indicated that in cellular environment, the main lipophilic oxo-containing species, phospholipids, could be one of the most representative targets for **AlL**.

**Table 1:** Binding ability of different oxo-containing substrates to **AlL**

Substrates	Lipophilicity	Net charge	Binding ability ^*a*^ ^,^^*b*^
ctDNA	Hydrophilic	>2	✗
CO_3_^2–^, SO_4_^2–^	Hydrophilic	–2	✗
PA, PG, OA, SDBS, CF-PE, NBD-PE, SDS	Lipophilic	–1	✓
M6P, H_2_PO_4_^–^	Hydrophilic	–1	✗
PE, PC	Lipophilic	0	✗

^*a*^✓ strong binding; ✗ weak binding.

^*b*^Binding ability was studied in HEPES buffer (pH 6.0, 10 mM) by UV-vis spectroscopy.

We thus focused our study on the titration of PG to **AlL** since it displayed the most remarkable spectral changes observed. When titrating PG (0–1.0 equivalent) to **AlL**, a progressive spectral broadening and intensity decrease were observed (Fig. 1a) in the UV-vis absorption spectrum, with isosbestic points at 462 and 623 nm. The fluorescence intensity of **AlL** also decreased dramatically to *ca.* 4% of the initial intensity after the addition of 1.0 equivalent of PG (Fig. 1c). The above spectral changes indicated the formation of aluminophosphate. The binding constant (*K*_b_) and binding site value (*n*) were determined through isothermal titration calorimetry in HEPES at pH 6.0 (Fig. S9†). In the presence of PG or PC, the binding site values are all about 1 (*n* = 1.02 ± 0.01 or *n* = 0.92 ± 0.09, respectively). However, the *K*_b_ of PG to **AlL** was estimated to be (1.17 ± 0.12) × 10^6^ M^–1^, which is two orders of magnitude higher that of electroneutral PC ((2.47 ± 0.58) × 10^4^ M^–1^). Due to the poor solubility of PA and PE in aqueous solution, we didn’t determine their binding constant to **AlL**. In addition, HR-ESI-MS gave an *m*/*z* value of 1227.70296 ([M + H]^+^), indicating the formation of a 1 : 1 adduct of **AlL-PG** (Fig. S10†). The kinetics of the binding of **AlL** to phospholipids was investigated by UV-vis spectroscopy in HEPES buffer (pH 6.0, 10 mM). Plotting the absorption at 390 nm or 588 nm *versus* the incubation time after adding PG to **AlL** showed that the binding of **AlL** to PG was a pseudo first-order reaction, with a binding rate constant of 0.8 s^–1^ at 25 °C (Fig. S11†).

Interestingly, when titrating PG to **AlL** from 1 to 180 equivalents, the UV-vis spectrum recovered to the initial spectral pattern, with a slight red shift from 587 to 593 nm (Fig. 1b). The fluorescence intensity also displayed an 11-fold increase (Fig. 1d). We tentatively ascribed this fluorescence increase to the fact that the formed **AlL-PG** adduct has dispersed into the lipid phase of the micelles formed by PG, since the concentration of PG is close to or larger than its critical micelle concentration (*ca.* 7 μM). To test the hypothesis that the fluorescence of **AlL-PG** increases significantly due to a microenvironmental change, we used CHCl_3_ to extract the *in situ* formed **AlL-PG** from its HEPES-buffered solution. As shown in Fig. S12,† compared to the fluorescence spectrum in HEPES buffer (pH 6.0, 10 mM), in the CHCl_3_ phase, the fluorescence intensity increased 100-fold. This sharp fluorescence enhancement thus demonstrated that **AlL-PG** exhibits more intense fluorescence in a non-polar environment than in a polar environment.

Thus, we proposed that the “tagging” of PG to **AlL** happens in two steps. In the first step, **AlL** binds to PG, which can be demonstrated by the large binding constant ((1.17 ± 0.12) × 10^6^ M^–1^) and binding rate constant (0.8 s^–1^). In the second step, the formed **AlL-PG** adduct disperses into the lipid phase of the phospholipid bilayers and the fluorescence is turned on.

To demonstrate the capability of **AlL** to bind to membrane vesicles, we prepared an artificial fluorescent liposome by adding 1% fluorescent CF-PE (1,2-dioleoyl-*sn-glycero*-3-phosphoethanolamine-*N*-(carboxyfluorescein)) to a mixture of POPC/POPG/cholesterol (molar ratio 10 : 1 : 2.5; mass ratio 20 mg : 2.2 mg : 2 mg) to mimic a membrane vesicle, where the aqueous interior is surrounded by a hydrophobic phospholipid bilayer.^48^ As shown in Fig. 1f and g, images from a confocal laser scanning microscope (CLSM) indicated that the red fluorescence of **AlL** overlapped well with the green fluorescence of CF-PE and both of them distributed homogeneously in the phospholipid bilayers. As shown in Video S1,† upon addition of **AlL** (1 μM), red fluorescent liposome circles appeared in 60 seconds due to the free diffusion of **AlL**. These results clearly suggested that **AlL** could bind to the hydrophobic phospholipid bilayers and turn on the fluorescence.

### Pulse-chase imaging of subcellular localization of **AlL**

**Fig. 2:**
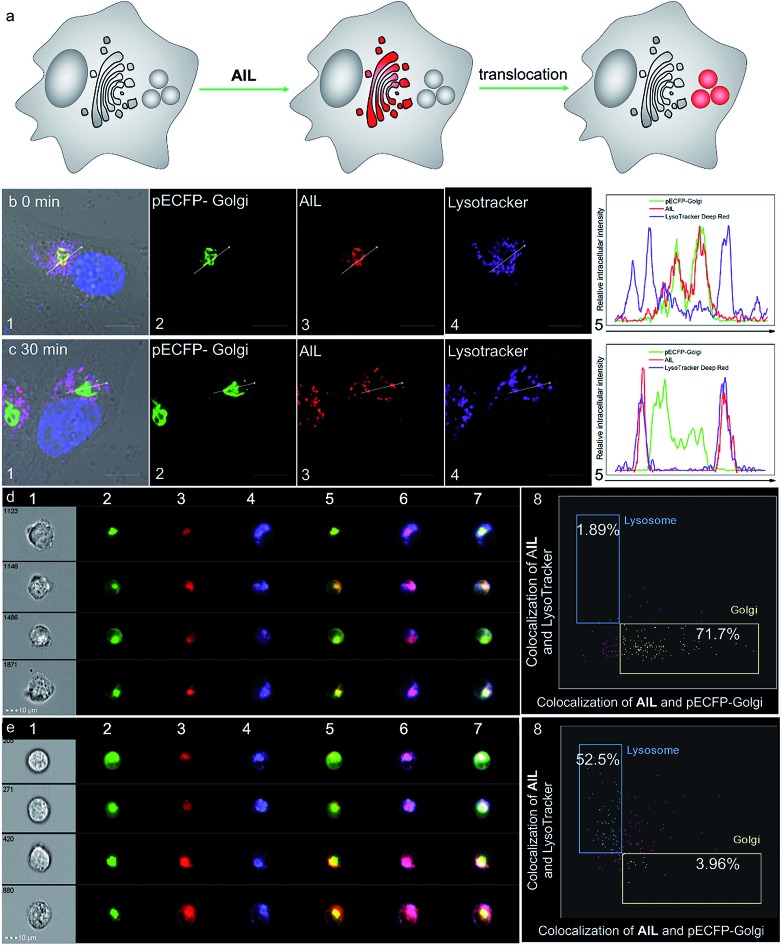
Colocalization assay of **AlL** (red) with pECFP-Golgi (green) and LysoTracker® Deep Red (purple) in HeLa cells. The nucleus was marked with Hoechst 33342 (blue). HeLa cells were pulsed with 1 μM of **AlL** for 5 min and monitored by confocal imaging at the indicated time. (a) Schematic illustration of the subcellular translocalization of **AlL**. Colocalization assay after (b) 0 min and (c) 30 min. (1) Merged images of (2–4) and DIC images; (2) images of pECFP-Golgi; (3) images of **AlL**; (4) images of LysoTracker® Deep Red; scale bar = 10 μm; (5) relative fluorescence intensity profile along the arrow in (b) and (c), respectively. ImageStream analysis of the intracellular localization of **AlL** at (d) 0 min and (e) 30 min using trans-Golgi marker and lysosome marker. (1) Bright-field images; (2) images of pECFP-Golgi; (3) images of **AlL**; (4) images of LysoTracker® Deep Red; (5) merged images of (2) and (3); (6) merged images of (3) and (4); (7) merged images of (2), (3) and (4); (8) bright detail similarity scores analysis of colocalization at 0 and 30 min, respectively. The percentage of colocalization **AlL** with the Golgi apparatus or lysosomes is indicated in each plot.

Prior to cell imaging, the cytotoxicity of **AlL** was assessed and low cytotoxicity was found, with up to 90% cell viability with an incubation of 2 μM complex for 48 hours (Fig. S13†). **AlL** was then used in conjunction with Hoechst 33342, pECFP-Golgi, and LysoTracker® Deep Red, which served as markers for the nucleus, Golgi apparatus and lysosomes, respectively, to determine the intracellular localization of **AlL**. HeLa cells were pulsed with 1 μM **AlL** for 5 min, washed, and cultured in fresh culture media again. As shown in Fig. 2a and b, **AlL** mainly distributed in the Golgi apparatus and gave merged bulk yellow fluorescence in the perinuclear region with a colocalization level of approximately 0.90 with pECFP-Golgi. In contrast, 30 min after the HeLa cells were pulsed with 1 μM **AlL**, **AlL** displayed merged punctuated pink fluorescence in the perinuclear region (Fig. 2a and c), with a colocalization level of approximately 0.90 with the lysosome tracker. The unbiased, large-scale statistical three-color image streams gave the same results as those from confocal imaging from the 50 000 cell event files (Fig. 2d and e). To quantify the association between **AlL** and either the Golgi apparatus or lysosomes, we employed a “similarity” algorithm contained in the IDEAS software that measures the degree of spatial colocalization of signals from different spectral channels.^49^,^50^ As shown in Fig. 2d and e, the amount of cells in which **AlL** colocalized with pECFP-Golgi contained within the double-positive gate significantly decreased from 71.7 to 3.96%, whereas colocalization of **AlL** with lysosomes markedly increased from 1.89 to 52.5%. Thus, the above results of the colocalization of **AlL** with the Golgi apparatus and lysosomes indicates that **AlL** is initially localized to the Golgi apparatus and later transits to the lysosomes in living cells. In addition, since **AlL** has a large two-photon absorption cross section (*ca.* 180 GM) at 840 nm, it can also be applied to image the Golgi apparatus using a two-photon excited fluorescence microscope (Fig. S14†).

### Dynamics of translocation of **AlL**

**Fig. 3:**
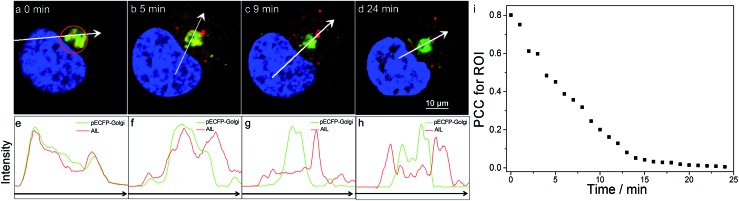
Dynamic monitoring of the translocation of **AlL** in HeLa cells pulsed with 1 μM **AlL** for 5 min. (a–d) Images at the indicated time; scale bar: 10 μm; (e–h) relative fluorescence intensity profiles along the arrows in (a–d), respectively. (i) Colocalization analysis of **AlL** to pECFP-Golgi in the region of interest (ROI) in (a) (red circle) with Pearson’s correlation coefficient (PCC).

We then visualized the dynamics of translocation of **AlL** from the Golgi apparatus to the lysosomes. After accumulating **AlL** in the Golgi apparatus for 5 min, HeLa cells were washed and imaged continuously. Initially, pECFP-Golgi and **AlL** completely colocalized, as indicated by the nearly identical intensity profiles (Fig. 3a and e). During the next 24 min, pECFP-Golgi and **AlL** separated and the degree of overlap in the intensity profiles decreased (Fig. 3b–d and f–h). After 24 min, pECFP-Golgi and **AlL** separated completely and the intensity profiles showed negligible overlap (Fig. 3d and h). The relative kinetics of **AlL** translocation was evaluated by plotting the change of the colocalization coefficient in the region of interest (Fig. 3a, red circle) *versus* the incubation time. As shown in Fig. 3i, the colocalization level of **AlL** to the Golgi apparatus decreased from 0.801 to 0.006.

From the time-series imaging, it only took 15 min for **AlL** to completely exit the Golgi apparatus and finally translocalize in the lysosomes. These dynamic intracellular transportation experiments clearly indicated that **AlL** could be used as an excellent tracker of vesicle trafficking from the Golgi apparatus to the lysosomes, which is one of the most important stages of lysosome biogenesis.^51^,^52^

### Transportation mechanism of **AlL** to the Golgi apparatus

**Fig. 4:**
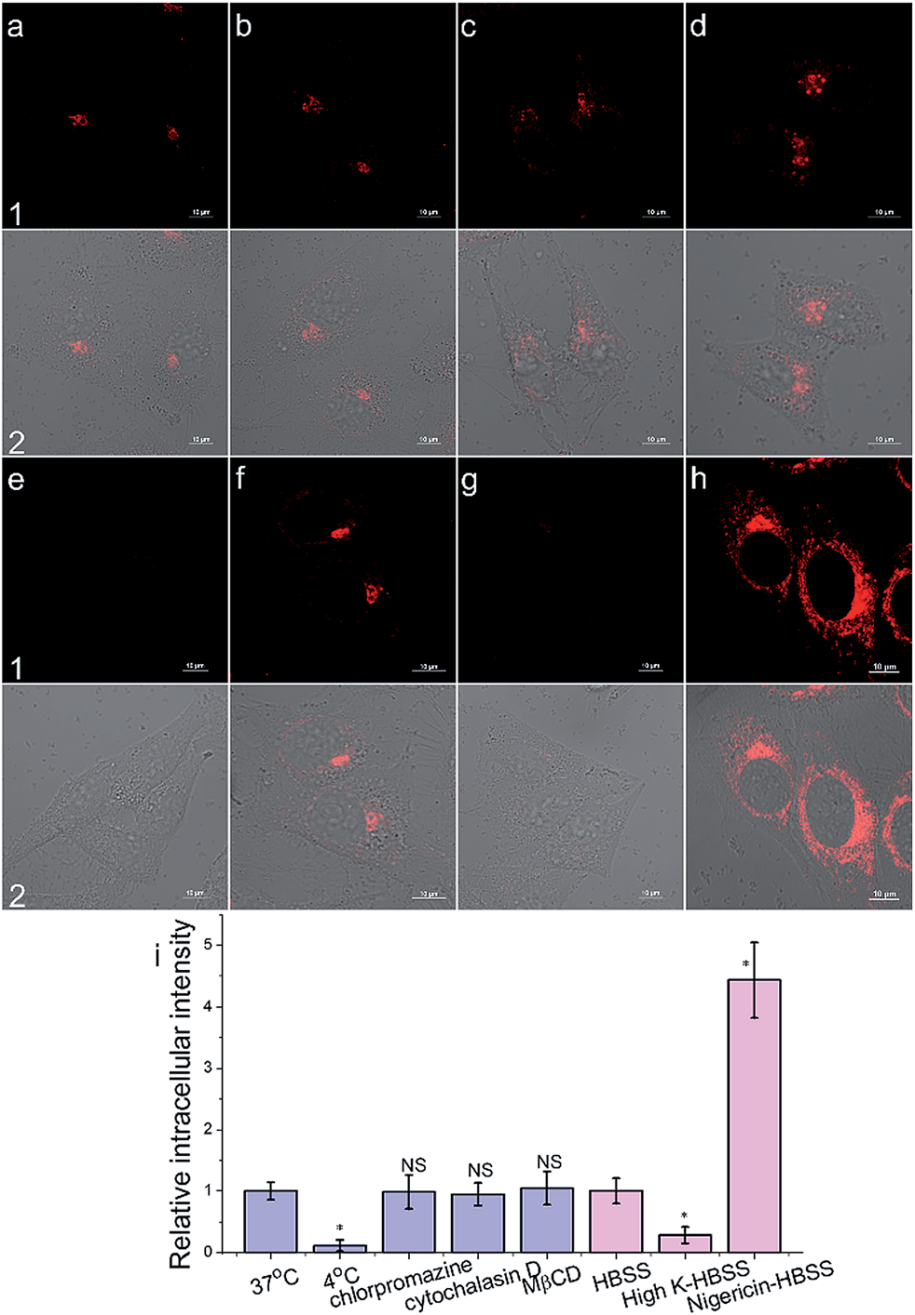
The cellular internalization mechanism of **AlL** (1 μM) into HeLa cells. (a–h) Images under different conditions: (a) 37 °C; (b) chlorpromazine; (c) cytochalasin D; (d) MβCD; (e) 4 °C; (f) HBSS; (g) high-K HBSS; (h) nigericin-HBSS. (1) Images of **AlL**; (2) merged images of (1) and Differential Interference Contrast (DIC) images; scale bar: 10 μm. (i) Histogram of the mean relative intracellular fluorescence intensities for intracellular uptake of **AlL** (*n* = 3, *P < 0.001 and NS = no significant difference).

To get an insight into the Golgi-apparatus-targeting ability of **AlL**, we investigated its cellular internalization pathway using CLSM focusing on the following three aspects: temperature, endocytosis and membrane potential. As shown in Fig. 4, only 10% intracellular fluorescence intensity was observed for **AlL** at 4 °C compared to that at 37 °C, indicating that **AlL** was internalized into cells *via* a temperature-dependent process. In the presence of different endocytosis inhibitors, chlorpromazine (inhibitor of clathrin-mediated endocytosis), MβCD (inhibitor of caveolae-mediated endocytosis), and cytochalasin D (inhibitor of macropinocytosis), the intracellular fluorescence intensities are close to the fluorescence of the control group where no inhibitor was used (98%, 104% and 95%, respectively), indicating that the uptake of **AlL** was not blocked by these three endocytosis inhibitors. Thus, we proposed that the membrane transport pathway of **AlL** was not related to endocytosis. The relationship between the uptake level and membrane potential was studied by depolarization or hyperpolarization of the plasma membrane. HeLa cells treated with high K^+^-HBSS (depolarization) displayed a remarkable decrease in intracellular fluorescence by 70%, while the cells treated with nigericin (hyperpolarization) showed an intracellular fluorescence increase of about 300%. These results demonstrated that the internalization of **AlL** is membrane potential-dependent. To summarise, the transmembrane pathway of **AlL** is most likely through membrane potential-dependent passive diffusion.

**Fig. 5:**
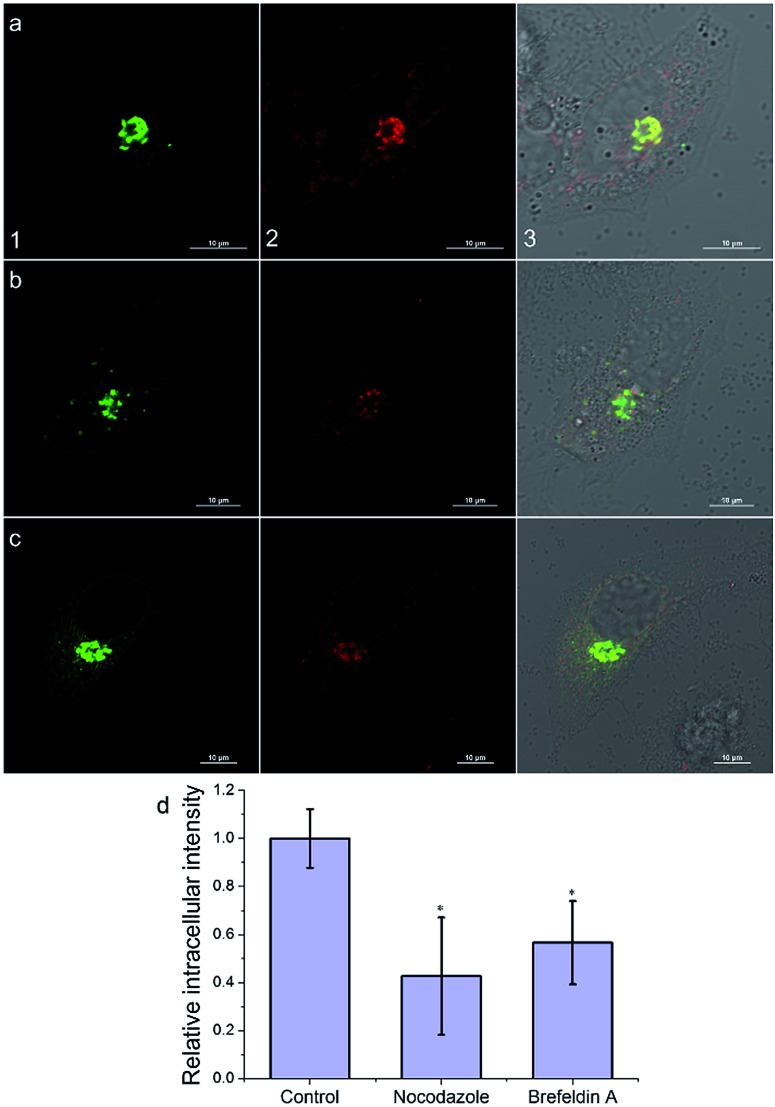
Intracellular transportation mechanism of **AlL** (1 μM) to the Golgi apparatus in HeLa cells. (a–c) Images under different conditions: (a) control; (b) nocodazole; and (c) brefeldin A. (1) Images of pECFP-Golgi; (2) images of **AlL**; (3) merged images of (1), (2) and Differential Interference Contrast (DIC) images; scale bar: 10 μm. (d) Histogram of the mean relative intracellular fluorescence intensities for intracellular uptake of **AlL** (*n* = 3 and *P < 0.001).

Then, we investigated the intracellular transport of **AlL** to the Golgi apparatus by treating cells with a microtubule-depolymerizing agent (nocodazole) and vesicle transport inhibitor (brefeldin A). As shown in Fig. 5, the intracellular fluorescence intensities of nocodazole- and brefeldin A-treated cells decreased to 43% and 56% of that of the control group, respectively. Therefore, we proposed that after **AlL** diffused into the cells, driven by the membrane potential, it was transported to the Golgi apparatus along microtubules through vesicle-trafficking.

### Mechanism of subcellular translocation of **AlL**

**Fig. 6:**
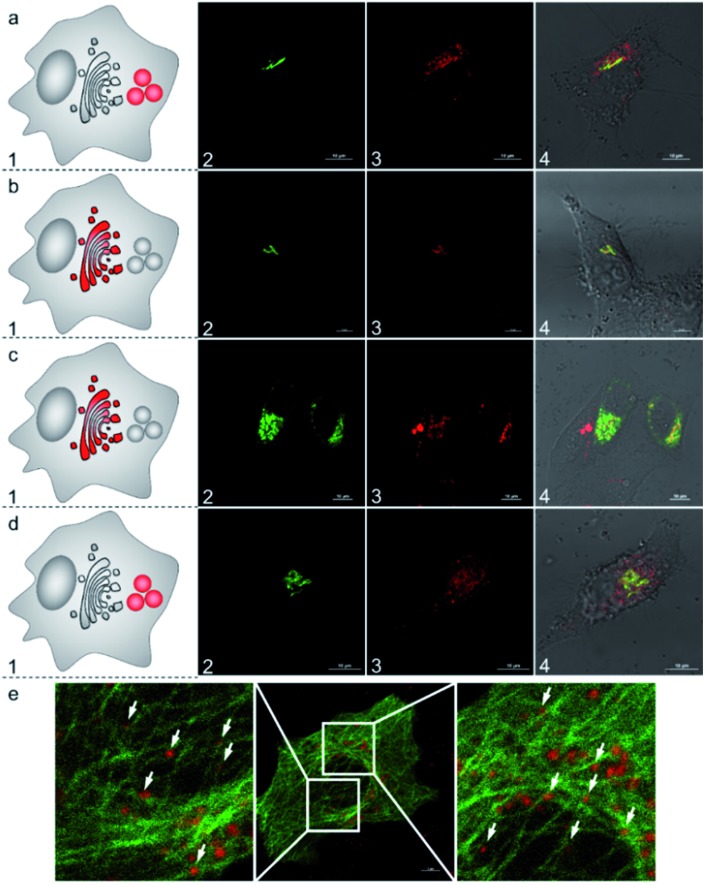
Subcellular translocation mechanism of **AlL** in HeLa cells pulsed with 1 μM **AlL** for 5 min. (a) At 37 °C; (b) at 19.5 °C; (c) with 60 μM nocodazole; (d) with 1 μg mL^–1^ cytochalasin D. Nocodazole and cytochalasin D were preincubated with the cells for 60 min and 30 min, respectively. (1) Images of Golgi ECFP; (2) images of **AlL**; (3) merged images of (1 and 2) and DIC images. (e) Confocal images of **AlL** (red) colocalized with EGFP-stained microtubules (green) in HeLa cells. The cells were pulsed with 1 μM **AlL** for 5 min and then incubated in fresh complete culture media for 15 min. Scale bar: 10 μm.

To further understand the subcellular translocation mechanism, we investigated the post-Golgi apparatus distribution of the **AlL** complex under different blocking conditions. As low temperature is known to drastically block vesicle trafficking pathways, we firstly examined the effect of temperature.^53^,^54^ HeLa cells were incubated with 1 μM **AlL** for 5 min at 37 °C, rinsed, and then cultured in fresh media at 19.5 °C for 30 min. As shown in Fig. 5b, the **AlL** red and pECFP-Golgi green fluorescence overlapped completely. As a control, we also set cells cultured with the **AlL** complex at 37 °C and no red fluorescence of **AlL** overlapped with the green fluorescence of pECFP-Golgi after 30 min (Fig. 6a). Second, we employed nocodazole and cytochalasin D, which destabilize the microtubules and actin network, respectively, to selectively block the translocation pathways.^55^ In 60 μM nocodazole-treated cells, the red fluorescence of **AlL** overlapped well with the green fluorescence of pECFP-Golgi, suggesting that subcellular transport of **AlL** was blocked with destabilized microtubules (Fig. 6c). When treated with cytochalasin D, however, the red **AlL** and the green pECFP-Golgi distributed separately, indicating no inhibition of **AlL** translocation through destabilization of the microfilaments (Fig. 6d).

To verify the microtubule-dependent vesicle transport from the Golgi apparatus to the lysosomes, we tracked the fluorescence of **AlL** in HeLa cells expressing β-tubulin EGFP, a marker for microtubules. As shown in Fig. 6e, at 15 min after being pulsed with **AlL** for 5 min, the red fluorescence of **AlL** was largely in close proximity to the green fluorescence of the β-tubulin EGFP-stained microtubules, suggesting that **AlL** moves along the microtubule structures. Altogether, these results showed that subcellular translocalization of **AlL** required an appropriate temperature and the presence of intact microtubules, suggesting that **AlL** underwent a membrane vesicle transport process from the Golgi apparatus to the lysosomes.

## Conclusions

Taken together, we report the first luminescent metal complex, **AlL**, which can target the Golgi apparatus and then track membrane vesicle trafficking from the Golgi apparatus to the lysosomes in living HeLa cells. **AlL** enters the cells through membrane potential-dependent passive diffusion and is transported to the Golgi apparatus *via* vesicle transportation along microtubules. Inside the territory of the Golgi apparatus, **AlL** has high potential for selective coordination to negatively mono-charged phospholipids, which together can further translocate to the lysosomes *via* membrane vesicle trafficking inside the cell. The intracellular pathway of **AlL** created a “fluorescent” vesicle flow from the Golgi apparatus to the lysosomes, which indicates the potential application of **AlL** as a fluorescent probe to help with the investigation of vesicle trafficking from the Golgi apparatus to the lysosomes. Such a strategy of employing a molecular fluorescent probe in vesicle trafficking would overcome the tedious workup of using fluorescent proteins. Ongoing work is aiming to pinpoint the exact phospholipid structures that bind to **AlL** in the cell environment and “carry” **AlL** to the designated organelles.

More broadly, as a case study, this work demonstrated a novel approach to probe intracellular molecular events with the aid of metal-induced Lewis acid reactivity. The uniqueness of the metal centers is of great importance in metal complexes to expand their biological applications as bioprobes and metal drugs, yet is commonly overlooked and rarely explored. This work opens up a new access to allow the interdisciplinary integration of knowledge of coordination chemistry into biological probes.

## Conflicts of interest

There are no conflicts to declare.

## Supplementary Material

Supplementary information

Supplementary information
