# Supplementary material for: A luminescent aluminium salen complex allows for monitoring dynamic vesicle trafficking from the Golgi apparatus to lysosomes in living cells
Source: Chem Sci. 2018 Jan 8;9(7):1931–9. doi: 10.1039/c7sc04498d (PMC5892405; doi:10.1039/c7sc04498d)
Supplement: Supplementary file 2 [file SC-009-C7SC04498D-s002.pdf]

Electronic Supplementary Material (ESI) for Chemical Science.
This journal is © The Royal Society of Chemistry 2018

A Luminescent Aluminium Salen Complex Allows for Monitoring Dynamic Vesicle Trafficking from Golgi Apparatus to Lysosomes in Living Cells

Juan Tang,[†] Hao-Yan Yin,[†] and Jun-Long Zhang[†]*

[†] Beijing National Laboratory for Molecular Sciences, State Key Laboratory of Rare Earth Materials Chemistry and Applications, College of Chemistry and Molecular Engineering, Peking University, Beijing 100871, P.R. China.

1. General experimental information

All solvents and chemicals for synthesis were purchased from Alfa Aesar and J&K and used as received without further purification, unless otherwise specified. Cellular imaging trackers and endocytosis inhibitors were purchased from Thermo Fisher Scientific. Phospholipids were purchased from A.V.T. (Shanghai) Pharmaceutical Co., Ltd and used as received without further purification.

The ^{1}H NMR spectroscopic measurements were carried out using a Bruker-400 NMR at 400 MHz with tetramethysilane (TMS) as internal reference. Electrospray ionization (ESI) mass spectra were performed on a Fourier Transform Ion Cyclotron Resonance Mass Spectrometer (Bruker, USA), positive-ion mode. The steady-state absorption spectra were obtained with an Agilent 8453 UV-vis spectrophotometer in 1 cm path length quartz cells. Single-photon luminescence spectra were recorded using fluorescence lifetime (Edinburgh Instrument FLS980) and steady state spectrophotometer (Hitachi F7000). Quantum yields of one photon emission of all the synthesized compounds were measured relative to the fluorescence of Rhodamine B (Φ = 0.65) in ethanol. The two photon fluorescence data was acquired using a Tsunami femtosecond Ti: Sapphire laser (pulse width $\leq$ 100 fs, 80 MHz repetition rate, tuning range 790 - 880 nm, Spectra Physics Inc., USA). FT-IR spectrum was taken on a Nicolet iN10 MX Fourier Transform Infrared Spectrometer. Isothermal Titration Calorimetry (ITC) was performed using MicroCal iTC200 (USA, Marvin). Confocal fluorescent images of living cells were performed using Nikon A1R-si Laser Scanning Confocal Microscope (Japan), equipped with lasers of 405/488/543/638 nm. Two photon fluorescence microscopy images were performed on a ZEISS LSM 510 META microscope system (Germany). ImageStream cytometry was measured by ImageStreamX markII (USA, Amnis).

2. Synthesis and characteristics

ZnSalen and **AlL** were fully characterized by ^{1}H NMR, high-resolution ESI-Mass (HR-ESI), and IR spectroscopes.

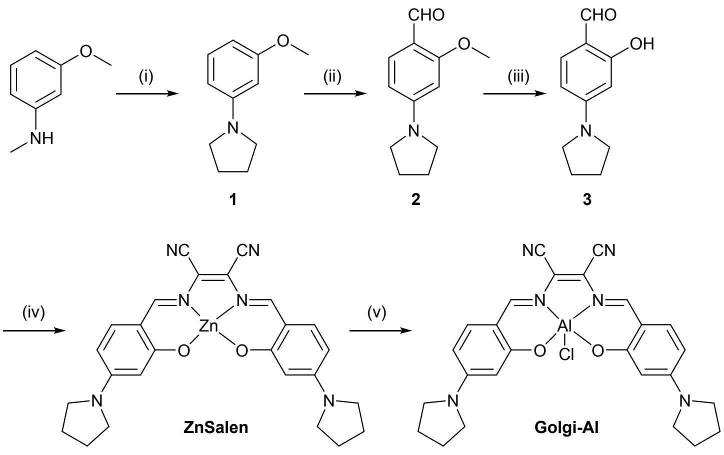

Synthetic route for **AlL**: (i) $Br(CH_2)_4Br$, $KHCO_3$, CH_3CN, 90℃, 12 h; (ii) $POCl_3$, DMF, 0℃ to r.t., 30 min; followed by hydrolysis in icy water, 30 min; (iii) BBr_3, DCM, -78℃ to r.t., 12 h; (iv) (v) 2,3-diaminomaleonitrile, $Zn(OAc)_2{\cdot}2H_2O$, ethanol, 90℃, 24 h; (v) $AlCl_3$, CH_3CN, 90℃, 24 h.

Compound 1

A reaction mixture of 3-methoxy-N-methylaniline (4.0 g, 32.0 mmol), 1,4-dibromobutane (3.8 g, 32.0 mmol) and $KHCO_3$ (4.4 g, 32.0 mmol) in 50 mL acetonitrile was refluxed under nitrogen for 12 h. After evaporation, the residue was extracted with CH_2Cl_2, washed and dried with anhydrous Na_2SO_4. Then the concentrated liquid was further purified by column chromatography to give yellow oil (2.33 g, 60%).

^{1}H NMR (300 MHz, $CDCl_3$): δ (ppm) 7.13 (1H, t, J = 8.1 Hz), 6.22 (2H, m), 6.11 (1H, t, J= 2.4 Hz), 3.80 (3H, s), 3.26 (4H, m), 1.99 (4H, m).

Compound 2

$POCl_3$ (1.3 mL, 13.9 mmol) was added slowly in anhydrous DMF (1.5 mL) in the ice-water bath and stirred for 30 min. Then compound **1** (2.5 g, 14.1 mmol) dissolved in DMF was added quickly. The mixture was slowly warmed to room temperature and stirred overnight. The reaction solution was poured into ice, stirred for a few minutes, and filtered to give light brown solid (1.8 g, 62%).

^{1}H NMR (300 MHz, CDCl3): δ (ppm) 10.13 (1H, s), 7.70 (1H, d, J = 8.7 Hz), 6.18 (1H, dd, J1 = 8.8 Hz, J2 = 1.4 Hz), 5.91 (1H, d, J = 1.7 Hz), 3.89 (3H, s), 3.44 (4H, m), 2.03 (4H, m).

Compound 3

2 (1.45 g, 7.1 mmol) was dissolved in 30 mL refreshed CH_2Cl_2, and boron tribromide (1.6 mL, 17.2 mmol) was added at -78°C. The mixture was warmed slowly to room temperature and stirred for 12 h. Cold methanol was added to quench extra boron tribromide. After evaporation and extraction, the residue was purified by column chromatography to give yellow oil (1.04 g, 77%).

^1H NMR (300 MHz, $CDCl_3$): δ (ppm) 11.69 (1H, s), 9.50 (1H, s), 7.28 (1H, d, J = 8.9 Hz), 6.17 (1H, dd, J1 = 2.1 Hz, J2 = 8.7 Hz), 5.97 (1H, d, J = 2.0 Hz), 3.38 (4H, t, J = 6.6 Hz), 2.04 (4H, m).

ZnSalen

A reaction mixture of compound **3** (76.4 mg, 0.4 mmol), 2,3-diaminomaleonitrile (21.6 mg, 0.2 mmol) and $Zn(OAc)_2 \cdot 2H_2O$ (43.8 mg, 0.2 mmol) in 10 mL ethanol was refluxed under nitrogen for 24 h. The system turned dark brown precipitate formed. After cooling to the room temperature and evaporating the solvent, the mixture was filtered and the solid was washed in turn by ethanol, ethyl acetate and petroleum ether. After dried under reduced pressure, compound **ZnSalen** was obtained as brown black solid (74.4 mg, 72%).

^1H NMR (400 MHz, d^6-DMSO): δ (ppm) 8.13 (2H, s), 7.18 (2H, d, J = 9.0 Hz), 6.15 (2H, dd, J1 = 1.9 Hz, J2 = 9.0 Hz), 5.71 (2H, d, J = 1.7 Hz), 3.36 (8H, m), 1.96 (8H, m).

HR MS (ESI^+, d^6-DMSO, FT-ICR): *m/z* calcd. for C26H35N6NaO2Zn ($[M+Na]^+$): 549.19324, found m/z: 549.19400.

FT-IR (KBr pellete, cm^{-1}): 2205 (C≡N), 1620 (C=N).

AlL

A reaction mixture of **ZnSalen** (76.4 mg, 0.4 mmol) and AlCl3 (43.8 mg, 0.2 mmol) in 10 mL acetonitrile was refluxed under nitrogen for 24 h. After cooling to the room temperature, the system was added with anhydrous ether. During this process, black precipitate could be observed. The mixture was filtered and the solid was washed in turn by mixture solvent of acetonitrile and

anhydrous ether (v/v=1/10). After dried under reduced pressure, compound **AlL** was obtained as brown black solid (28.8 mg, 56%).

^{1}H NMR (400 MHz, d^{6}-DMSO): δ (ppm) 8.15 (1H, s), 7.42 (1H, d, J = 11.1 Hz), 6.31 (1H, d, J = 11.1 Hz), 5.89 (1H, s), 3.44 (4H, s), 1.98 (4H, s).

HR MS (ESI^{+}, DMSO, FT-ICR): *m/z* calcd. for C28H30AlN6O3S ($[M\text{-}Cl\text{-}+DMSO]^{+}$): 557.19100, found m/z: 557.18926.

FT-IR (KBr pellete, cm^{-1}): 2216 (C≡N), 1620 (C=N).

^{1}H NMR of AlL

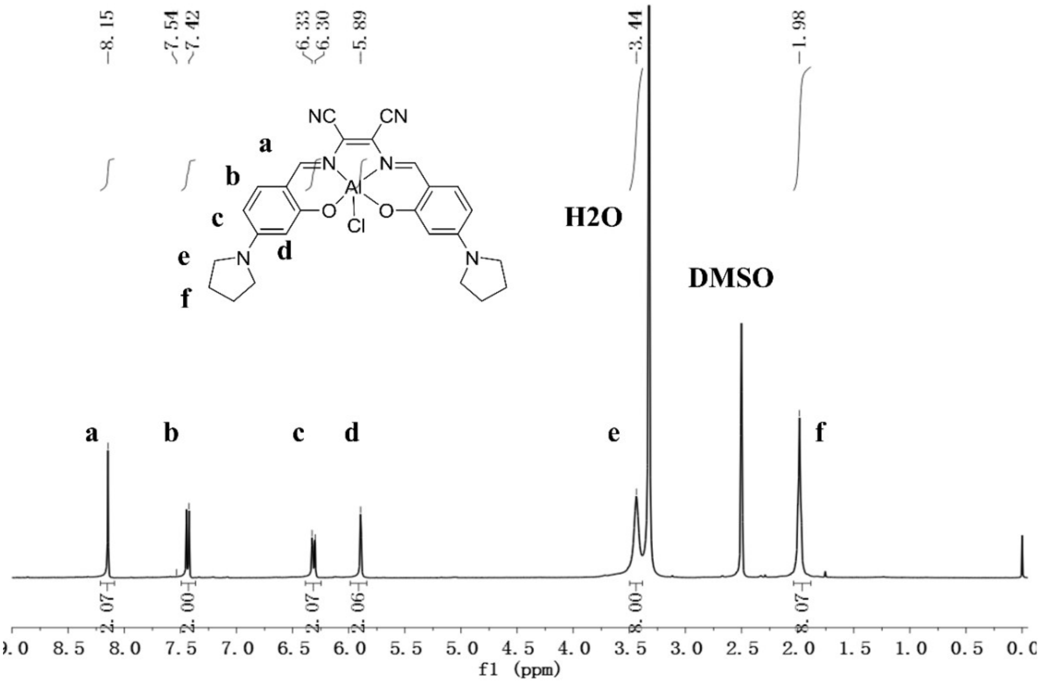

HR-MS of AlL

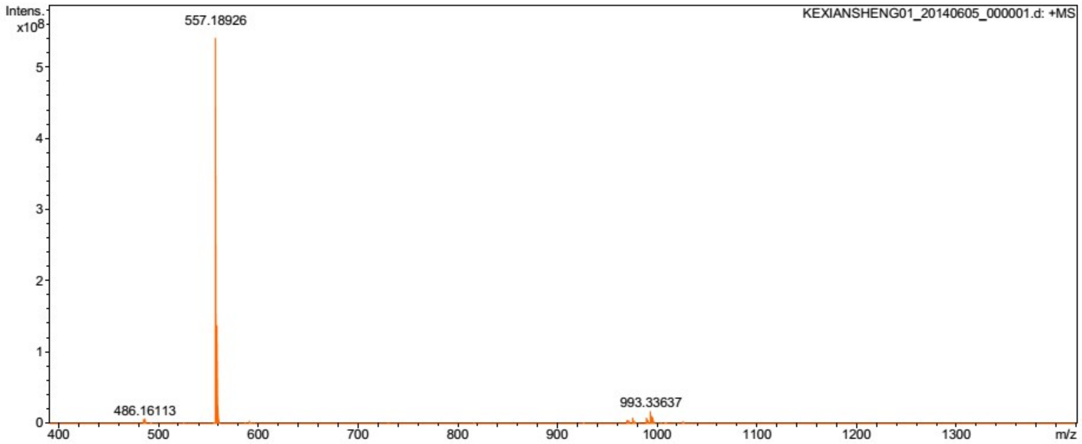

Meas. m/z	I	Formula	m/z	err [mDa]	err [ppm]	mSigma	rdb	e¯ Conf	N-Rule
557.18926	553918080	C 28 H 30 Al N 6 O 3 S	557.19100	1.74	3.12	51.4	17.5	even	ok

IR spectra of AlL

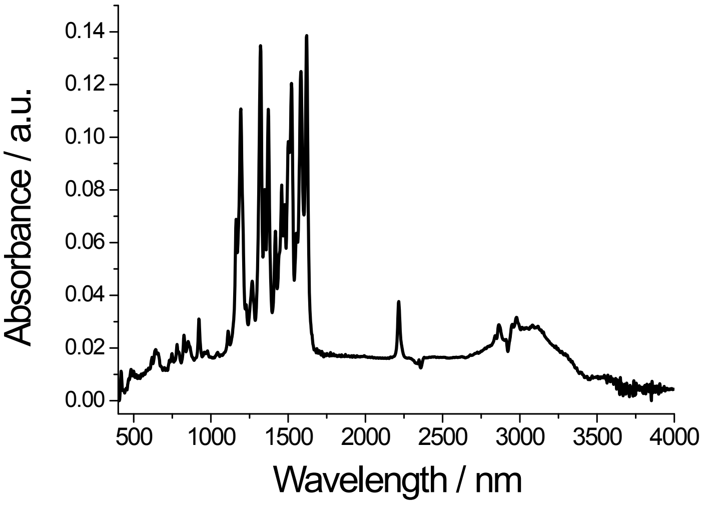

3. Quantum yield Determination

Quantum yield of one photon emission of **AIL** was measured with Rhodamine B as reference (Φ=0.65). The one photon fluorescence measurements were performed in 1cm quartz cells with 20 μM compound in DMSO on a fluorescence lifetime and steady state spectrophotometer (Edinburgh Instrument FLS920) equipped 450 W Xenon light, slits 2.5 × 2.5. The values of fluorescence quantum yield, Φ (sample), were calculated according to equation as following:

$$\frac{\Phi_{sample}}{\Phi_{ref}} = \frac{OD_{ref} \cdot I_{sample} \cdot d_{sample}^2}{OD_{sample} \cdot I_{ref} \cdot d_{ref}^2}$$

Φ: quantum yield;
I: integrated emission intensity;
OD: optical density at the excitation wavelength;
d: the refractive index of solvents, d_{DMSO}=1.478, $d_{ethanol}$=1.361.

4. Measurement of the two-photon absorption cross section

The two-photon absorption spectra of **AIL** were determined over a broad spectral region (750 nm - 860 nm) by the typical two-photon induced fluorescence method relative to Rhodamine B as standard.[1] The two-photon fluorescence data were acquired using a Tsunami femtosecond Ti: Sapphire laser (pulse width ≤ 100 fs, 80 MHz repetition rate, tuning range 750 - 860 nm Spectra Physics Inc., USA). The two-photon fluorescence measurements were performed in a 1cm quartz cell with 10 μM sample dissolved in DMSO and the excitation power density is set to be 200 mW. The two-photon absorption cross section (δ_{sample}) was calculated at every 10 nm wavelength from 750 nm to 860 nm according to equation as following:

$$\delta_{sample} = \delta_{ref} \cdot \frac{\Phi_{ref} \cdot C_{ref} \cdot I_{sample} \cdot d_{sample}}{\Phi_{sample} \cdot C_{sample} \cdot I_{ref} \cdot d_{ref}}$$

δ: Two-photon absorption cross section of the reference (Rhodamine B), which was read out from the previous literature.
Φ: Quantum yield of sample and reference;
I: integrated emission intensity;
C: concentration of each sample;
d: The refractive index of solvents, d_{DMSO}= 1.478, $d_{ethanol}$ = 1.361.

5. pH effect of AIL

pH effect on **AIL** was monitored by UV-vis spectra and fluorescence spectra. 20 μM **AIL** was

dissolved in Britton-Robinson buffer of different pH (4.0-10.0).

6. Binding ability of AIL to phospholipids

Binding ability was assessed by UV-vis spectra, fluorescence spectra and ITC. The titration was carried out in Hepes buffer (pH 6.0) containing 20 μM **AIL**. For the ITC experiments, the titrate (POPG) and the substrate (**AIL**) were prepared in the same mixture solution (DMSO: Hepes) to avoid the solvent dilution heat.

7. Lop P of AIL-PG adduct

Equal amounts of n-octanol and Hepes buffer (pH 6.0, 10 mM) were thoroughly mixed by an oscillator for 24 h. The mixture was then left to separate for another 24 h to finally yield aqueous and octanol phase, each saturated with the other. We first dissolved **AIL** in HEPES buffer at 20 μM level, and then added 20 μM of PG to in situ form **AIL**-PG (C_o), followed by mixing with equal amounts of octanol (saturated with water) and shaken again as described above. After separation, the final concentrations of compounds in Hepes buffer corresponded to C_w. The final concentration in 1-octanol corresponded to $C_{oil.}$ [5]

$$P = \frac{C_{oil}}{C_{water}} \quad (\textit{Equation.}\ \mathrm{S3})$$

8.

8. GUV preparation

GUVs were prepared by gentle hydration method.[3] POPC/POPG/FI-PE/cholesterol (molar ratio 10:1: 0.1:2.5; mass ratio 20 mg:2.2 mg:0.2 mg:2 mg) were dissolved in 10 mL mixed solvent of chloroform and methanol (volume ratio 3:1) in a glass round-bottom flask. The solvent was evaporated with rotary evaporator. The round-bottom flask was left inside a vacuum chamber overnight to remove residue solvent. The lipid film was under a stream of water saturated nitrogen for half an hour to prehydrate the film. About 10 mL of GUV buffer (PBS containing 200 mM sucrose, pH 7.4) was then gently transferred into the tube and against the tube walls. The round-bottom flask was left inside the 37℃ water bath overnight. **AIL** solution (2 mM stocking in DMSO) was added into the well prepared GUVs solution, and the mixture was stirred softly for half an hour. To get confocal images, the vesicles were placed in a PBS (pH 7.4) containing 200 mM glucose environment. The effect of gravity could fix the vesicle to make for a snapshot.

9. Cell culture

HeLa cells were incubated in complete medium (Dulbecco's modified Eagle's Medium, supplemented with 10% fetal bovine serum (FBS) and 1% penicillin-streptomycin) at 37°C in atmosphere containing 5% CO_2.

10. CCK-8 assay

HeLa cells were seeded in flat-bottomed 96-well plates, 1×10^4 cells per well, with 200 μL complete culture media for 24 h. After washed with PBS for three times, the cells were incubated with different concentrations of **LD-TPZn**. All stock solutions were prepared in DMSO (2 mM) and diluted with complete media. After cultured for 24 h, the cells were washed with PBS (pH 7.4) three times. 10 μL Cell Counting Kit-8 (CCK-8) solution and 90 μl PBS (pH 7.4) were added per well simultaneously. After 2 hours, the absorbance at 450nm was read by 96-well plates reader. The viability of Hela cells was calculated by the following equation:

$$CV = (As-Ab) / (Ac-Ab) \times 100\%$$

CV stands for the viability of cells, As, Ac and Ab stand for the absorbance of cells containing **LD-TPZn**, cell control(0 μM **LD-TPZn**) and blank control (wells containing neither cells nor **LD-TPZn**).

11. Pulse-chase confocal imaging of AlL

A stock solution of **AlL** in chromatographic grade, anhydrous DMSO was prepared as 2 mM. The solution was diluted to a final concentration of 1 μM by complete growth medium. LysoTracker® Deep Red and Hoechst 33342 were prepared as 1 mM DMSO stock solution and 10 mg mL^{-1} water solution, respectively. The stock solution was diluted to the working concentration in complete medium (72 nM and 10 μg mL^{-1} Hoechst 33342).

Hela cells were grown to about 80% confluency and then reseeded in 24-well plates; cells were transfected with 0.8 μg pECFP-Golgi plasmid, using LipfectamineTM 2000 according to manufacturer's instruction. After 6 h, HeLa cells were digested with trypsin and replaced onto 0.1 mM poly-D-lysine coated glasses in complete media and the cells were incubated for 24 h.

HeLa cells were incubated with LysoTracker® Deep Red for 30 min and Hoechst 33342 for 5 min, respectively. After washed with PBS (pH 7.4) for three times, cells were incubated with 1 μM **AlL** for 5 min. Then cells were washed with PBS (pH 7.4) for three times and further incubated in fresh complete growth medium. Confocal imaging was carried out at 0 min or 30 min The cells Images were taken under conditions as follows: 60× immersion lens with a resolution of

1024×1024 and a speed of 0.25 frame per second; 80% laser power for Hoechst 33342, 405 nm excitation wavelength and 425 to 475 nm detector slit; 50% laser power for pECFP-Golgi, 488 nm excitation wavelength and 500 to 530 nm detector slit; 100% laser power for **AlL,** 543 nm excitation wavelength and 552 to 617 nm detector slit; 20% laser power for LysoTracker® Deep Red, 639 nm excitation wavelength and 662 to 737 nm detector slit;. Differential interference contrast (DIC) and fluorescent images were processed and analyzed using ImageJ. The Pearson's Coefficient was calculated by ImageJ.

For ImageStream flow cytometry analysis, cells are detached from culture to produce a cell suspension of 2×10^6 cells in 2 mL PBS (pH 7.4). Images were acquired on the Imagestream® imaging cytometer (Amnis Corp., Seattle, WA). At least 10,000 images were collected for each sample. Cells were collected with the laser scatter image in channel 1, the pECFP-Golgi image in channel 2 (green, 488 nm excitation wavelength), the **AlL** image in channel 10 (red, 561 nm excitation wavelength), LysoTracker® Deep Red image in channel 11 (purple, 642 nm excitation wavelength), and the bright field image in channel 5 (white). Post-acquisition spectral compensation and data analysis was performed using IDEAS® image analysis software package (Amnis Corp.) based on a novel algorithm, the similarity bright detail score (SBDS), which is designed to quantitate the degree of co-localization of two cell surface proteins. SBDS quantifies the degree of similarity between any two channel images on a pixel- by-pixel and cell-by-cell basis. The SBDS is calculated in a three-step process: 1) determination of the opening residue image for each channel image; 2) calculation of a non-mean normalized Pearson's correlation coefficient; 3) log transformation of the correlation coefficient to give roughly Gaussian distributions, the mean of which is the SBDS. The SBDS is calculated for each of ~10,000 cells per data file and averaged, thus allowing for a rigorous and statistically significant analysis of each cell population. Similarity algorithm is now widely used for studying the intracellular localization and trafficking.

For time-series imaging of the dynamic of the subcellular translocation, HeLa cells were incubated with 1 μM **AlL** for 5 min and then incubated in fresh complete growth medium without phenol red. XYTime images were obtained using a confocal microscope equipped with a live cell incubator.

12. Mechanism of cellular internalization pathway

The cellular uptake of luminescent metal complexes is primarily examined using confocal microscopy. In the temperature effect assay, cells were placed at 4 °C for 30 minutes, and then incubated with **AlL** for 5 min at 4 °C. For endocytosis mechanism investigation, various endocytosis inhibitors including chlorpromazine (inhibitor of clathrin-mediated endocytosis, 10 μg·mL^{-1}), MβCD (inhibitor of caveolae-mediated endocytosis, 10 mM), cytochalasin D (inhibitor of macropinocytosis, 5 μg·mL^{-1}) were applied to cells for 30 minutes. Then medium containing both inhibitors and complex was used for incubation for 5 min. In the membrane potential effect

assay, HeLa cells were washed three times with either HBSS (containing 5.8 mM K^+) or high K^+-HBSS (containing 170 mM K^+). Some of the cells in HBSS were pretreated with 10 μM nigericin for 30 min at 37°C. The cells were incubated with 1 μM **AlL** for 5 min at 37°C in one of the following solutions: HBSS, HBSS with nigericin (to hyperpolarize the cells), or high K+-HBSS (to depolarize the cells). For the transportation to Golgi apparatus, HeLa cells were pretreated with nocodazole (60 μM) or Brefeldin A (50 μM) for 1 h, and then were treated with medium containing both inhibitors and complex for 5 min.

After incubation, the cells were rinsed, and the extent of uptake was analyzed by confocal imaging and dealt with ImageJ. Images were taken under conditions as follows: 60× immersion lens with a resolution of 1024×1024 and a speed of 0.5 frame per second, 543 nm excitation wavelength and 552 to 617 nm detector slit, 100% laser power for dye. Differential interference contrast (DIC) and fluorescent images were processed and analyzed using ImageJ.

13. Mechanism of subcellular translocation

The cellular translocation is primarily examined using a Nikon A1R-si Laser Scanning Confocal Microscope (Japan). In the temperature effect assay, cells were incubated with 1 μM **AlL** for 5 min and further incubated in fresh complete growth medium for 30 min at 37℃ or 19.5℃. In the cytoskeleton effect assay,

To disrupt the function of microtubules, HeLa cells were incubated with 60 μM nocodazole for 2 h. To disrupt the function of microfilaments, HeLa cells were incubated with media containing 5 $\mu g \cdot mL^{-1}$ cytochalasin B for 30 min. Then cells were incubated with 1 μM **AlL** for 5 min and further incubated in fresh complete growth medium for 30 min at 37℃. The inhibitors were maintained in the cell culture throughout the experiments.

14. Two-photon confocal microscopy imaging

Two photon fluorescence microscopy images were performed on ZEISS LSM 510 META microscope system. HeLa cells seeded on 0.17 mm-thick cover glasses were incubated with 1 μM **AlL** for 5 min. After washed with PBS (pH 7.4) for three times, two-photon imaging was carried out. The microscopy settings were as follows: 60× objective, a resolution of 1024×1024, 840 nm excitation wavelength, 80% laser power (50 mW).

Table S1. Photophysical data of **AlL** [a]

λ_{abs}(nm)	ε^{b} (*$10^4 M^{-1}cm^{-1}$)	λ_{em} (nm)	Φ^{c}	τ (ns)
394, 443, 595	4.92, 1.73, 5.98	643	0.42	4.3

[a] **AlL** was dissolved in DMSO (20 μM)； ε: extinction coefficients. Φ: Quantum yield, which was measured using Rhodamine B in ethanol as reference; τ: fluorescence lifetime.

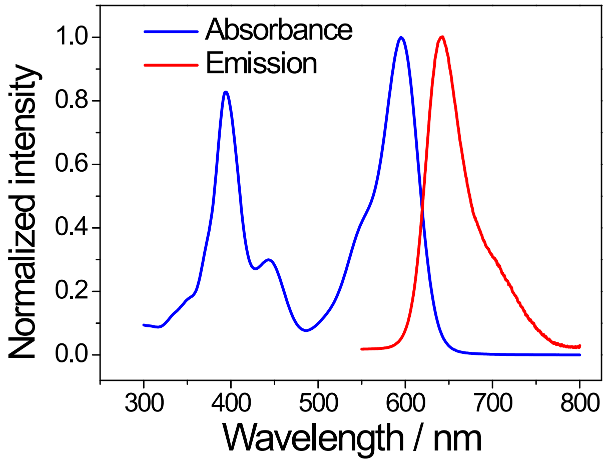

Figure S1. Normalized UV-*vis* and fluorescence spectra of **AlL** in DMSO, λ_{ex} = 415 nm.

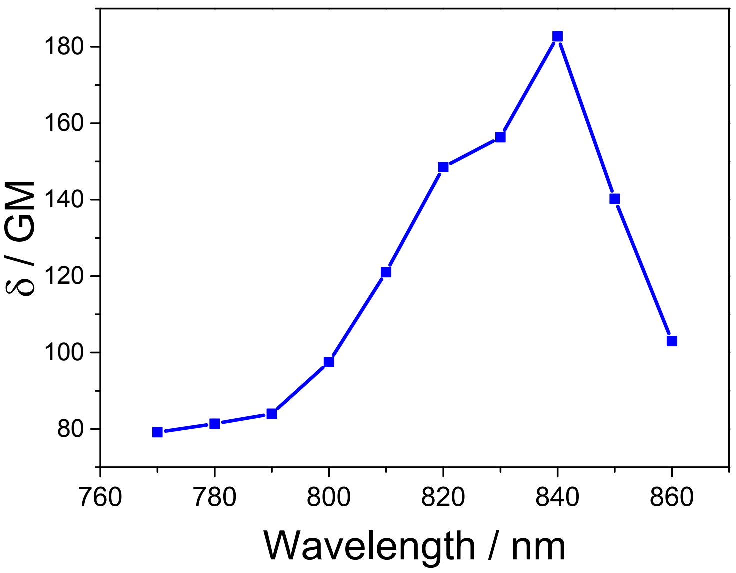

Figure S2. Two-photon absorption cross section of **AIL** in DMSO using Rhodamine B as a reference.

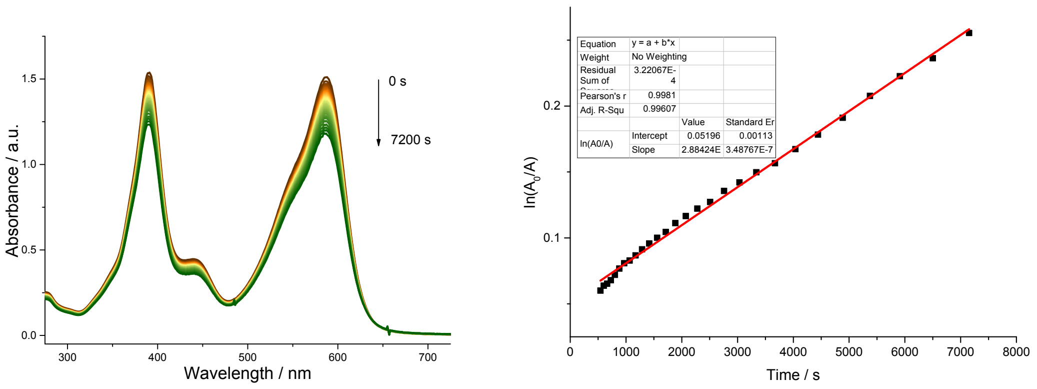

(a) (b)

Figure S3. (a) Hydrolysis of **AlL** in Hepes buffer (pH 6.0, 10 mM) monitored by UV-vis spectra and (b) plot of the absorption maximum decay at 588 nm versus of time.

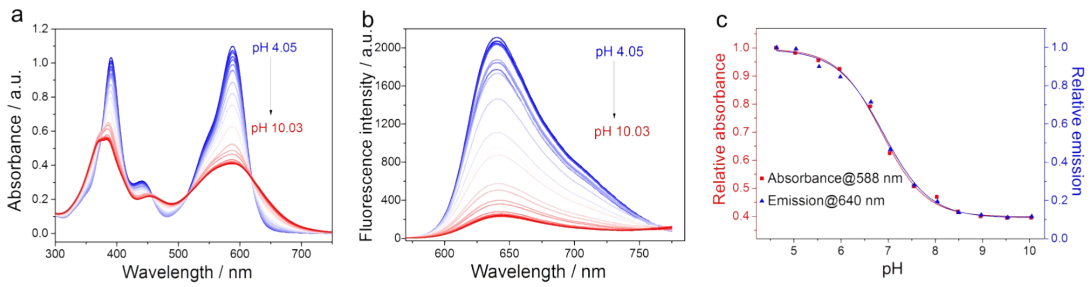

Figure S4. pH effect on **AIL** (20 μM). (a) UV-*vis* and (b) fluorescence spectra in Britton-Robinson buffer at different pH, λ_{ex} = 415 nm. (c) Changes of relative absorbance at 588 nm and fluorescence intensity at 640 nm of **AIL** along with different pH.

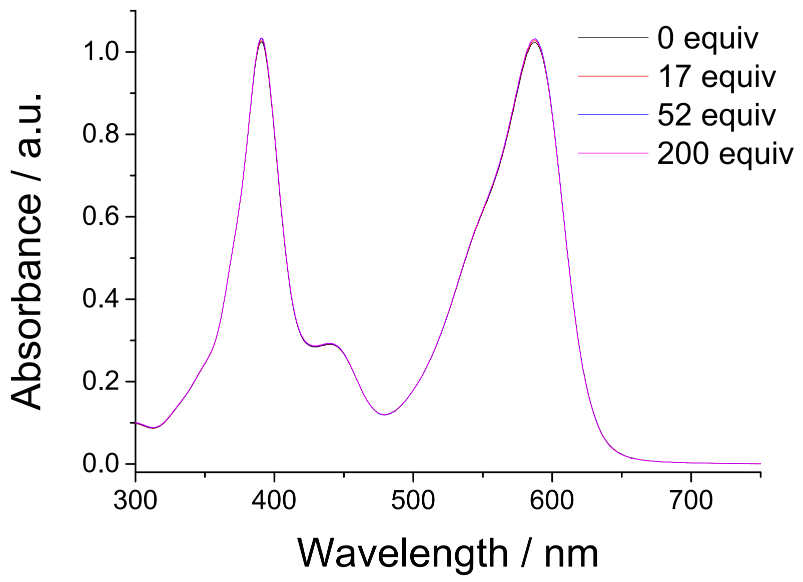

Figure S5 UV-vis titration of **AlL** (20 μM) with PC in Hepes buffer (pH 6.0).

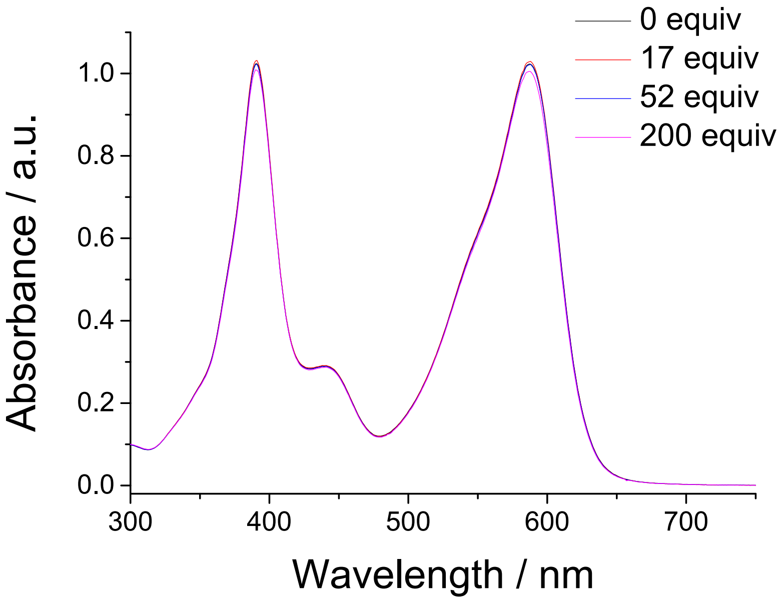

Figure S6 UV-vis titration of **AlL** (20 μM) with PE in Hepes buffer (pH 6.0).

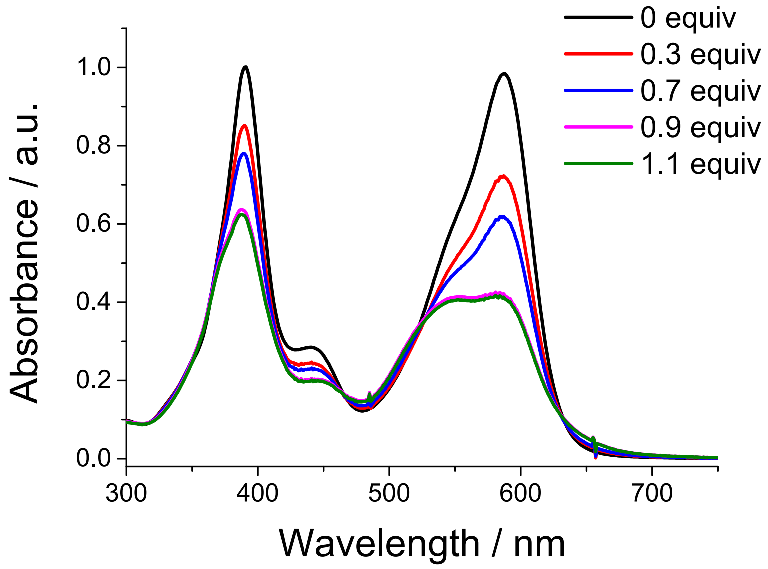

Figure S7 UV-vis titration of **AlL** (20 μM) with PA in Hepes buffer (pH 6.0).

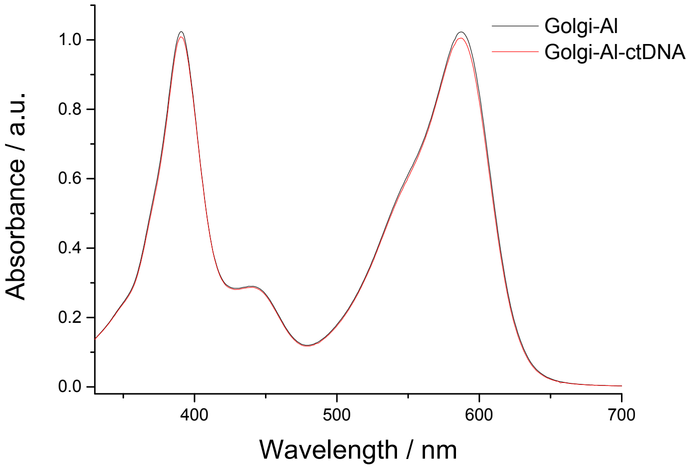

Figure S8 UV-vis titration of **AlL** (20 μM) with ctDNA (40 μg/mL) in Hepes buffer (pH 6.0).

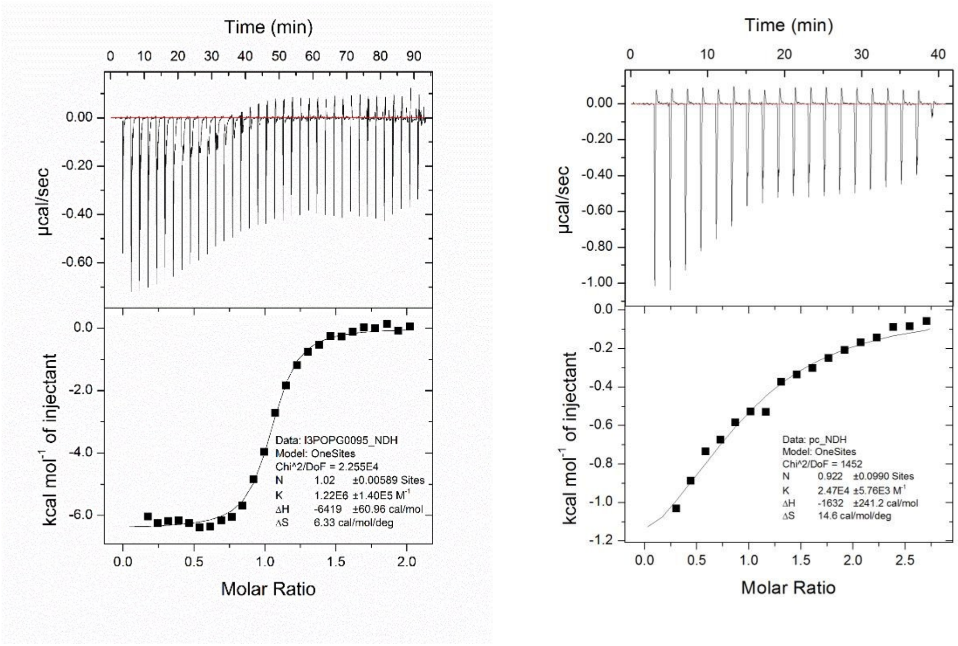

(a) (b)

Figure S9. ITC Titration Binding Curves, where K is the binding constant and N is the number of binding sites. (a) PG binding to **AlL**; (B) PC binding to **AlL**.

(1)

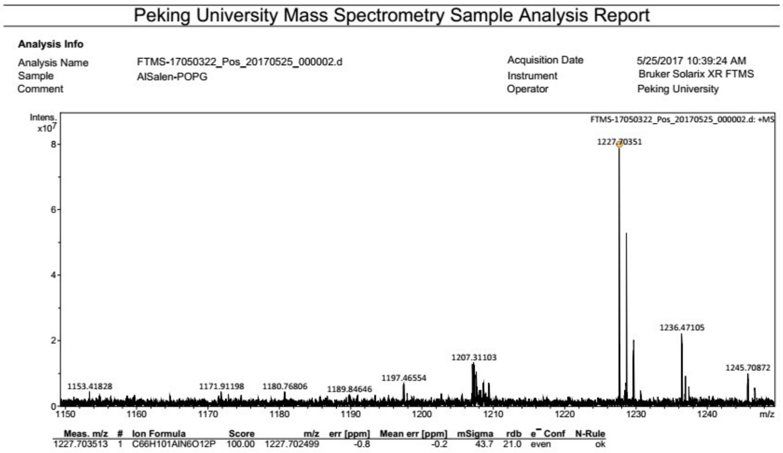

Figure S10 High-resolution electrospray ionization mass of the 1:1 adduct of **AlL**-phosphatidylglycerol.

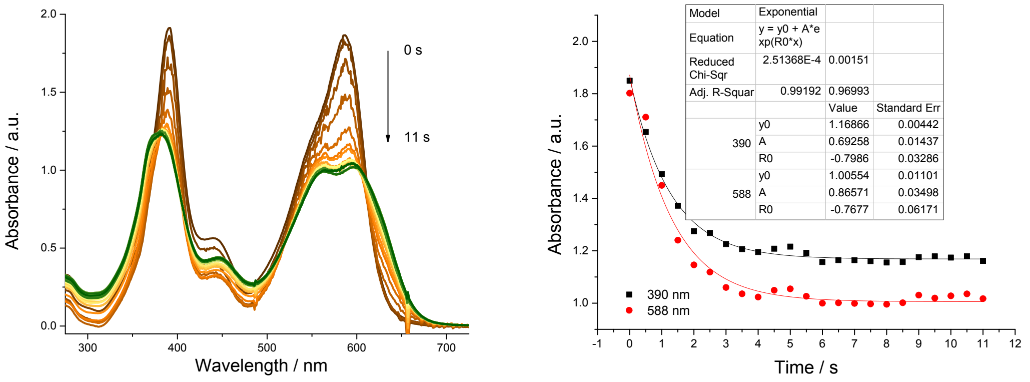

(a) (b)

Figure S11 (a) The kinetics of the binding of **AlL** (20 μm) to PG (20 μm) monitored by UV-vis spectra in Hepes buffer (pH 6.0, 10 mM). (b) Plot about the absorption at 390 nm or 588 nm versus the incubation time.

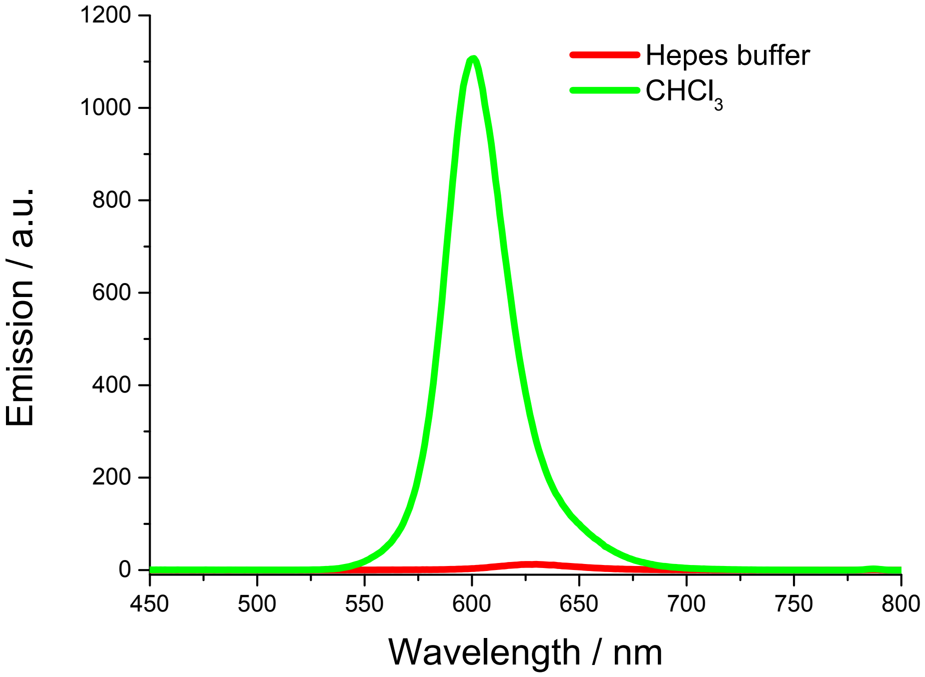

Figure S12 Fluorescence of **AlL**-PG adduct in Hepes buffer (pH 6.0, 10 mM) and $CHCl_3$.

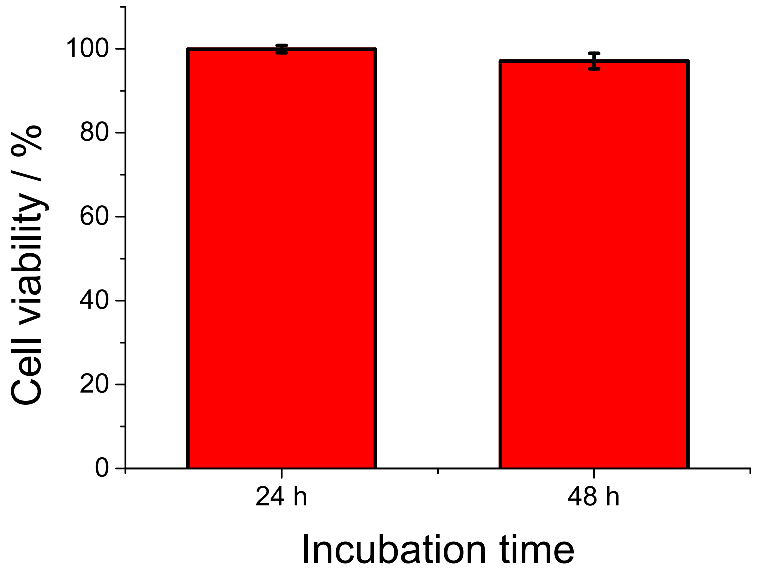

Figure S13. Cytotoxicity in HeLa cells after incubation with 2 μM of **AlL** for 24 h and 48 h.

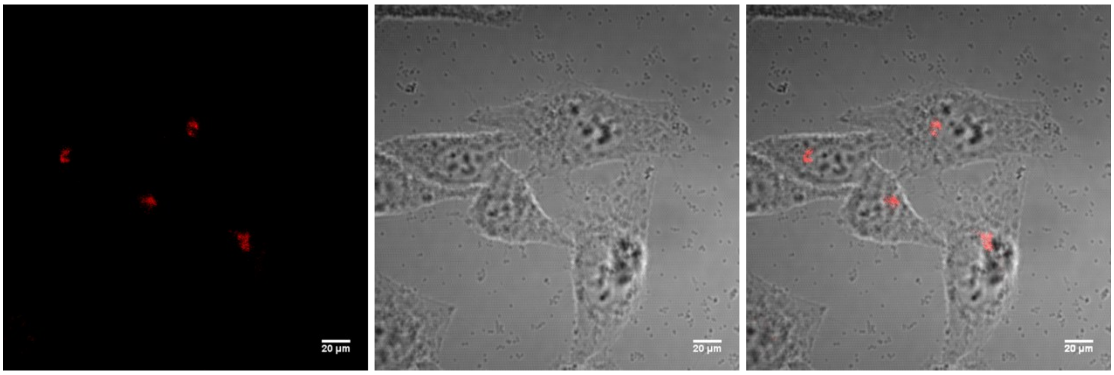

Figure S14. Two-photon fluorescence imaging of LDs in HeLa cells incubated with **AlL** (1 μm) for 5 min.

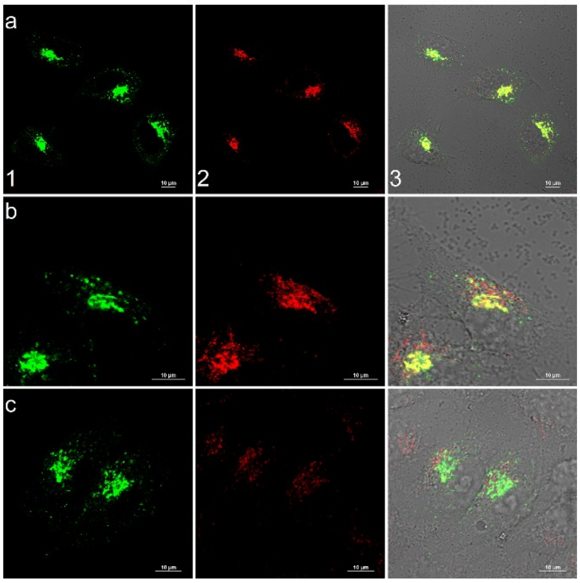

Figure S15. Images of **AlL** (1 μM) with MPR-GFP-expressed HeLa cells at indicated coincubation time: (a) 5 min; (b) 15 min; (c) 30 min; (1) images of MPR-GFP; (2) images of **AlL**; (3) merged images of (1), (2) and Differential Interference Contrast (DIC) images; scale bar: 10 μm.
